# The Effect of Local Melatonin Application on Bone Fracture Healing in Rat Tibias

**DOI:** 10.3390/medicina61010146

**Published:** 2025-01-16

**Authors:** Murat Tanrisever, Bahar Tekin, Umit Koray Can, Ozmen Istek, Erhan Cahit Ozcan, Ibrahim H. Ozercan, Turker Gelic, Serkan Dundar

**Affiliations:** 1 Department of Surgery, Faculty of Veterinary Medicine, Firat University, Elazig 23119, Türkiye; tgelic@firat.edu.tr; 2 Department of Peridontology, Faculty of Dentistry, Firat University, Elazig 23119, Türkiye; dtbahartekin@gmail.com (B.T.); sdundar@firat.edu.tr (S.D.); 3 Turkish Jockey Club Elazig Racecourse Horse Hospital, Elazig 23350, Türkiye; koraycan23@gmail.com; 4 Department of Nursing, Faculty of Health Medicine, Mus Alparslan University, Mus 49250, Türkiye; o.istek@alparslan.edu.tr; 5 Department of Esthetic, Plastic and Reconstructive Surgery, Faculty of Medicine, Firat University, Elazig 23119, Türkiye; ecozcan@firat.edu.tr; 6 Department of Pathology, Faculty of Medicine, Firat University, Elazig 23119, Türkiye; ozercanih@yahoo.com

**Keywords:** bone formation, fracture healing, local melatonin, rat

## Abstract

*Background and Objectives:* This study aimed to histologically evaluate the effects of local melatonin application at different doses on bone fracture healing. *Materials and Methods:* Thirty rats were divided into three groups, with ten rats in each group. In the control group (*n* = 10), a fracture line was created in the tibial bones, and fracture osteosynthesis was performed without any additional procedure. In the local melatonin dose 1 (MLT D-1) group (*n* = 10), a fracture line was created in the tibial bones, and 1.2 mg of lyophilized powder melatonin was applied locally before fracture osteosynthesis. In the local melatonin dose 2 (MLT D-2) group (*n* = 10), a fracture line was created in the tibial bones, and 3 mg of lyophilized powder melatonin was applied locally before fracture osteosynthesis. After a 12-week healing period, all subjects were sacrificed, and tibial bones were collected for histomorphometric analysis. *Results:* The percentage of bone formation was significantly higher in the MLT D-1 and MLT D-2 groups than in the control group. There was no statistically significant difference between the MLT D-1 and MLT D-2 groups. *Conclusions:* In conclusion, the study demonstrated that local melatonin application supports bone fracture by increasing bone formation, although different doses of melatonin did not lead to significant variations in fracture healing.

## 1. Introduction

Bone tissue is composed of osteoblasts, osteoclasts, osteocytes, stem cells, minerals like calcium and phosphate, and a collagen matrix that provides both compressive and tensile strength [1]. Due to the balance between bone resorption by osteoclasts and bone formation by osteoblasts, this structure is continually remodeled throughout life. Bone remodeling is a dynamic process involving the formation of new bone tissue formation by osteoblasts and the degradation of existing bone tissue by osteoclasts [1,2]. Fracture healing is a complex mechanism in which cells interact with growth factors that promote cell division and differentiation, while the extracellular matrix provides structural support for new bone tissue. Various factors, such as age, bone type, nutritional status, existing bone diseases, and medications, can influence this process. The key cells involved in fracture healing include inflammatory cells, chondrocytes, stem cells, osteoblasts, osteoclasts, pericytes, and endothelial cells [3]. Among these, osteoblasts and osteoclasts play a central role in bone formation and resorption [4].

The bone remodeling process is regulated by growth factors, cytokines, prostaglandins, leukotrienes, and systemic hormones, such as parathyroid hormone, vitamin D, calcitonin, growth hormone, estrogen, androgen, and melatonin [5]. Melatonin, one of the most potent known antioxidants, is produced in the pineal gland but can also be synthesized locally in bone marrow and other tissues [6]. Due to its high lipophilic properties, melatonin can easily pass through cell membranes and intracellular compartments [7]. It exhibits both antioxidant and anti-inflammatory effects, with high doses exhibiting anti-inflammatory effects, including reducing free radicals and proinflammatory cytokines [8]. Melatonin has been shown to regulate bone cells and gene expression related to osteoblasts [9,10]. Its effects on bone metabolism occur through three primary mechanisms: supporting osteoblast differentiation and activity, inhibiting osteoclast differentiation, and scavenging free radicals [11].

The balance between osteoprotegerin (OPG) and receptor activator of nuclear factor kappa-B ligand (RANKL) is critical for osteoclastogenesis (bone resorption). Melatonin inhibits osteoclastogenesis by targeting RANKL with OPG, potentially reducing bone loss. By modulating other signaling pathways affecting osteoblastogenesis, melatonin acts at multiple levels in bone remodeling [12,13].

By promoting osteoblastic differentiation, melatonin may support fracture healing. Studies on preosteoblast MC3T3-E1 cells and rat osteoblast-like osteosarcoma 17/2.8 cells have shown that melatonin increases the production of bone marker proteins such as bone sialoprotein, alkaline phosphatase (ALP), osteocalcin, and osteopontin. These proteins are indicators of bone formation and mineralization, and so increasing the levels of these markers using melatonin may positively impact bone healing processes [14,15].

During bone injury, inflammation and oxidative stress are common. With its strong antioxidant and anti-inflammatory properties [16], melatonin inhibits the activity of oxygen radicals during fracture healing and supports the healing process by regulating the activity of antioxidant enzymes. In a previous study, greater bone fusion was observed on day 28 of the healing process in the melatonin-treated fracture group compared to the untreated fracture group. These findings suggest that melatonin plays an important role in supporting fracture healing [17].

The effects of melatonin on angiogenesis (the formation of blood vessels), which precedes osteogenesis, are also significant for bone regeneration [18]. New bone formation (NBF) depends on the presence of blood vessels to provide mineral elements and allow the migration of angiogenic and osteogenic cells to the target areas [19]. Studies have shown that melatonin treatment increases vascular endothelial growth factor (VEGF) levels during granulation tissue formation, accelerating the angiogenic process. These findings suggest that melatonin may have beneficial effects in bone defect repair and vascular injuries [20]. Given these effects, numerous studies have demonstrated melatonin’s significant role in bone formation and stimulation [21,22,23,24].

In this study, we proposed that the local application of melatonin during bone fracture repair procedures may enhance NBF. Therefore, we aimed to evaluate the histomorphometric effects of two different doses of local melatonin application on new bone regeneration in the treatment of bone fractures in rat tibias.

## 2. Materials and Methods

### 2.1. Animals and Study Design

This study was approved by Firat University (protocol number: 19746, date: 13 November 2023) Local Animal Experiments Ethics Committee and conducted at the Firat University Experimental Research Center, adhering strictly to the Helsinki Declaration guidelines. A total of 30 one-year-old, 300–350 gr Sprague Dawley female rats were used. To ensure standardization in the study, rats in the same estrus period were identified by vaginal smear and included in the study. The animals were housed in temperature-controlled cages, subjected to a 12/12 h light/dark cycle, and given ad libitum access to food and water throughout the experiment. The sample size was determined by power analysis (8% bias), with a type 1 error (α) of 0.05, a type 2 error (β) of 0.20 (power = 0.80), and a minimum of 10 animals per group. Rats were randomly divided into 3 groups: control group (*n* = 10), local melatonin dose 1 (MLT D-1) group (*n* = 10), and local melatonin dose 2 (MLT D-2) group (*n* = 10). In the control group, a fracture was created in the diaphyseal region of the right tibial bones using a steel disk, and fracture osteosynthesis was performed using Kirschner wire, with no additional procedure. In the MLT D-1 group, the same procedure was performed, but 1.2 mg of lyophilized powder melatonin (Sigma-Aldrich, St Louis, MO, USA) was applied topically to the intramedullary region at the fracture line before osteosynthesis. Similarly, in the MLT D-2 group, 3 mg of lyophilized powder melatonin (Sigma-Aldrich, St. Louis, MO, USA) was applied topically to the intramedullary region at the fracture line before osteosynthesis.

This study, which investigated the effects of local melatonin application on fracture healing, was designed based on findings from earlier research. The study by Dundar et al. [21], conducted on rabbits, served as a reference. Comparable results were obtained in studies by Munoz et al. [23] which examined local melatonin application in dogs.

### 2.2. Surgical Procedures

All surgical procedures were performed under sterile conditions. General anesthesia was induced using intramuscular injections of 10 mg/kg xylazine (Rompun, Bayer, Germany) and 40 mg/kg ketamine (Ketasol, Richter Pharma, Wels, Austria). Before the surgical procedure, the surgical area was shaved and cleaned with a povidone–iodine solution. Following asepsis and antisepsis protocols, an approximately 2.5 mm long incision was made over the crest of the tibial bone using a No. 15 scalpel blade. After elevating the soft tissues and periosteum, the diaphyseal region of the tibial bone was exposed. A bicortical bone cut was made under serum perfusion with a rotary disk. The bone were stabilized with Kirschner wires, and the soft tissues in the surgical area were repositioned and sutured. Since the diaphysis of the tibia bone has a flat structure, the bone thicknesses in this region are equal. Standardization was ensured by performing a bicortical bone cut, which completely separated the bone fragments while maintaining uniform thickness in the diaphyseal region. No fatal or nonfatal complications were detected during and after the experimental procedures.

Post-surgery, rats received intramuscular antibiotics (40 mg/kg cefazolin sodium) and analgesics (0.1 mg/kg tramadol hydrochloride). 

### 2.3. Histomorphometric Analysis

Tibial bones preserved in zinc–formalin were kept in tap water for 2–3 h, dehydrated in an alcohol series (70%, 80%, 90%, and absolute) for 24 h, and cleared in xylene solution for 2 × 30 min. The tissues were then embedded in paraffin blocks. Tissue sections 4–6 μm thick were prepared using a microtome (Leica RM2265; Leica, Wetzlar, Germany) and these sections were placed in an oven set at 60 °C and kept for 3–4 h. Then, they were passed through xylene for 2 × 30 min and through 96%, 90%, and 70% alcohol series for 10 min, respectively. Sections were then passed through distilled water for 2 × 5 min and subjected to hematoxylin staining for 8 min. They were then washed in running water for 5 min. After removing excess water from the sections, they were stained in eosin for 5 min. The sections were passed through an alcohol series of increasing concentrations and kept in xylene for 2 × 30 min. For histological analysis, the sections were coverslipped with Entellan^®^ (catalog no. 107961, Sigma-Aldrich, St. Louis, MO, USA) and examined with an Olympus light microscope (Olympus BX43, Tokyo, Japan).

After a 12-week healing period, all subjects in each group were sacrificed. Tibial bones were preserved in 10% formaldehyde for 72 h and subsequently demineralized in 10% formic acid. Following demineralization, the bones were dried, embedded in paraffin, and prepared for histological sectioning. Samples 6 μm thick were obtained using a rotary microtome and stained with hematoxylin–eosin. NBF was assessed by analyzing histological sections with a trinocular light microscope equipped with a camera and an image analysis system [25,26].

To quantify NBF, the area of callus tissue at the fracture site was measured. The regions within the callus devoid of bone were subtracted from the total callus area to calculate the bone-filled area. The NBF percentage (%) for each sample was determined by dividing the total bone-filled area by the total callus area. Histological analyses were performed in a blinded manner.

### 2.4. Statistical Analysis

IBM SPSS version 22 was used for statistical analysis. Data conformity to normal distribution was verified using Shapiro–Wilk and Kolmogorov–Smirnov tests. Since the data followed a normal distribution, a one-way analysis of variance (one-way ANOVA) was used to evaluate differences among groups, and the Tukey HSD (Honestly Significant Difference) test was applied to identify the source of differences. Results were presented as mean ± standard deviation, with significance set at *p* < 0.05. Statistical analysis was performed blindly by a researcher who did not know which group the data belonged to.

## 3. Results

**Figure 1:**
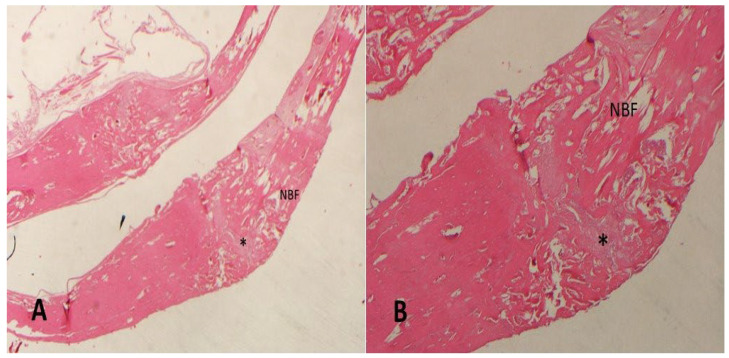
Decalcified histologic images of the control group ((**A**) 20× and (**B**) 40× magnification; hematoxylin–eosin). NBF: new bone formation; *: fibrosis.

The study was completed over 12 weeks with 10 rats in each group. No infections, wound dehiscence, or mortality occurred. Histomorphometric analysis results are shown in Table 1. The control group exhibited the lowest bone formation percentage (48.7 ± 6.62%) (Figure 1).

**Figure 2:**
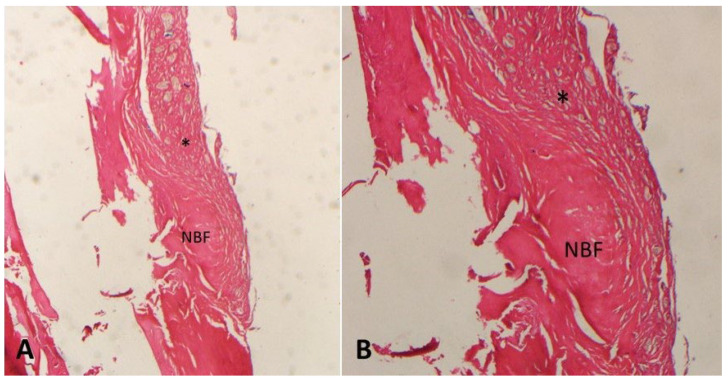
Decalcified histologic images of the MLT D-1 group ((**A**) 20× and (**B**) 40× magnification; hematoxylin–eosin). NBF: new bone formation; *: fibrosis.

**Figure 3:**
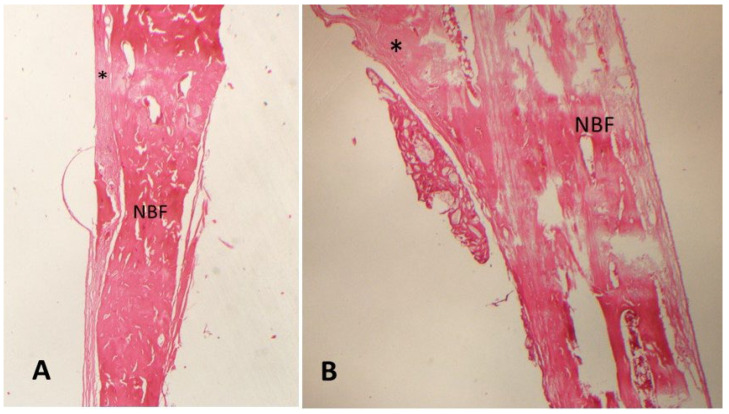
Decalcified histologic images of the MLT D-2 group ((**A**) 20× and (**B**) 40× magnification; hematoxylin–eosin). NBF: new bone formation; *: fibrosis.

The highest bone formation percentages were in the MLT D-1 group (58.1 ± 4.82%) (Figure 2) and the MLT D-2 group (59.2 ± 3.58%) (Figure 3). The bone formation percentages in the MLT D-1 and MLT D-2 groups were significantly higher than in the control group, but no statistically significant difference was found between the MLT D-1 and MLT D-2 groups.

## 4. Discussion

In this study, the percentage of NBF was 58.1% in the MLT D-1 group, 59.2% in the MLT D-2 group, and 48.7% in the control group. While there was no statistically significant difference between the two different doses of local melatonin in fracture bone healing, the difference between the groups that received melatonin and the control group was statistically significant.

Fracture repair requires cells that enable the calcification of bone tissue, an organic matrix, and inorganic substances [27]. Osteoblasts, osteocytes, osteoclasts, and osteogenic precursor cells in bone tissue play a critical role in bone formation and structural integrity, along with type I collagen fibers and various non-collagenous components [27,28]. Bone remodeling occurs through the interaction of hormones, cytokines, growth factors, and other biomolecules [29].

Melatonin is a key hormone regulating bone formation and absorption. Acting as a local growth factor, melatonin directly influences osteoblasts by accelerating the maturation of preosteoblasts, resulting in a significant increase in bone matrix production and calcification rates [30,31]. By binding to receptors on preosteoblasts, melatonin reduces the differentiation time of osteoblasts from 21 to 12 days, an effect mediated by indole through membrane receptors [12]. Additionally, melatonin enhances ALP activity, leading to elevated levels of bone morphogenetic protein 2 human (BMP-2) and osteopontin (OPN), which boost bone mineral density, bone neoformation [31,32].

The initial stage of bone healing, known as the inflammatory phase, is characterized by ischemia, clot formation, reperfusion injury, and the infiltration of inflammatory cells. During this phase, free oxygen radicals produced by neutrophils can damage cell membranes, hindering fracture healing through lipid peroxidation [24,33]. Research has shown that melatonin mitigates these effects by suppressing free radical activity and regulating antioxidant enzyme levels in this process [17,20,34]. In the subsequent proliferative phase, characterized by cell differentiation, collagen accumulation, angiogenesis, and granulation tissue formation [33], melatonin supports osteoblast proliferation and differentiation by promoting collagen accumulation [20]. Furthermore, melatonin has been shown to regulate angiogenesis during fracture healing [18,22]. For example, Zheng et al. demonstrated that systemic melatonin treatment in a rat tibial defect model significantly increased angiogenic markers, including VEGF, angiopoietin-1, and angiopoietin-2 [35].

The connection between melatonin and bone metabolism is well established, with numerous studies highlighting its role in promoting bone cell activity and formation [5,24,36]. Our study investigated the effects of local melatonin application on bone fracture healing in a rat model. The findings revealed a significant increase in bone formation in the MLT D-1 and MLT D-2 groups compared to the control group, supporting the hypothesis that melatonin enhances fracture healing through its anti-inflammatory and antioxidant properties.

Various administration routes and formulations of melatonin have been explored in animal studies, including local application of the lyophilized hormone [21,37], systemic administration via intraperitoneal injection [38,39], and oral administration through gavage [40]. For example, systemic intraperitoneal melatonin administration has been shown to promote osteoblastic differentiation in MC3T3-E1 cells and enhance fracture healing via the PDGF/AKT signaling pathway [15]. It was found that systemic melatonin application supports fracture healing by promoting the osteoblastic differentiation of mesenchymal stem cells and NPY/NPY1R signaling [41]. Additionally, it was shown to increase bone mineral density and bone formation by supporting osteogenesis [34]. Altintepe et al. reported that systemic melatonin application inhibited alveolar bone resorption and increased osteoblastic activity by reducing RANKL expression and inflammatory cell infiltration [42].

Despite its benefits, systemic melatonin application requires high doses, increasing the risk of side effects. Consequently, topical application is often preferred [43]. However, there is limited research on the effect of different local melatonin doses on fracture healing. Our study aims to address this gap and contribute valuable insights to the literature in this field.

In our study, we applied lyophilized powder melatonin locally at two doses: 1.2 mg and 3 mg. The percentages of NBF were 58.1 ± 4.82% in the group that received 1.2 mg of melatonin and 59.2 ± 3.58% in the group that received 3 mg. No statistically significant difference was found between the groups, suggesting that melatonin is effective within a specific dose range and that higher doses may not confer additional benefits. These findings offer valuable insights for determining the optimal effective dosage of melatonin.

In a similar study by Dundar et al. [21], lyophilized powder melatonin was also administered locally at doses of 1.2 mg and 3 mg, and NBF around the implant was evaluated histomorphometrically. Unlike our findings, their results showed a significantly higher percentage of bone formation in the group that received 3 mg of melatonin, indicating a potential discrepancy between studies. Other studies involving the local application of 1.2 mg melatonin reported increased NBF and trabecular bone area density around implants in melatonin-treated groups compared to controls [23,44]. Similarly, a study using 3 mg of locally applied melatonin supported these studies, showing significantly higher implant-related bone formation in the melatonin-treated group [45]. Clinical studies have further demonstrated the positive effects of local melatonin application on bone formation around implants. For example, patient groups that received 1.2 mg of melatonin gel before implant placement exhibited better stability and reduced marginal bone loss in implants [36,46,47]. In a study investigating the effects of local melatonin application on bone defect treatment in osteoporotic rats, groups treated with 3 mg pure melatonin or 1.5 mg melatonin combined with 1.5 mg xenogeneic bone graft showed increased NBF and osteopontin expression, along with a regulated inflammatory response, compared to the groups that did not receive melatonin [48]. These findings underscore the positive effects of locally applied melatonin on bone healing mechanisms, and the results of our study confirm this effect.

The present study has some limitations. First, the molecular mechanisms linking local melatonin application and bone metabolism could not be fully elucidated due to the methodology used in this study. Second, while in vivo studies are crucial for understanding the pathways underlying bone fracture healing, their results can only be used to extrapolate the corresponding pathways in humans. Third, we could not evaluate the long-term success of bone fracture healing in this study.

## 5. Conclusions

In conclusion, our study demonstrated that local melatonin application positively impacts bone fracture healing, although no statistically significant differences were observed in bone formation percentage between the tested doses. Within the limitations of our study, local melatonin application may be considered a supportive treatment method for bone healing. Further research is needed to clarify the relationship between melatonin and bone healing mechanisms.

## Figures and Tables

**Table 1:** New bone formation (NBF) ratios of the groups.

Groups	N	Mean (NBF) (%)	Std. Dev.	*p* *
Control	10	48.7	6.62	0.000
MLT D-1 ^a1^	10	58.1	4.82	
MLT D-2 ^a2^	10	59.2	3.58	

* One-way ANOVA *p* = 0.000. ^a1,a2^: Tukey HSD. ^a1,a2^: Statistically significantly different compared with control. ^a1^: 0.001; ^a2^: 0.000. A statistically significant difference was not detected between the MLT D-1 and MLT D-2 groups (*p* > 0.05).

## Data Availability

Data are contained within the article.
